# Integrating in-silico and experimental validation approaches to unveil the therapeutic mechanism of naringenin against breast cancer

**DOI:** 10.1038/s41598-025-19931-6

**Published:** 2025-10-15

**Authors:** Suhail Ahmad Mir, Basharat Ahmad Bhat, Laraibah Hamid, Umme Hani, Zahrah Ali Ahmed Asiri, Nasir Nisar, Riyaz Ali M. Osmani, Nazima Haider, Ashraf Dar, Ghulam Nabi Bader

**Affiliations:** 1 Department of Pharmaceutical Sciences, University of Kashmir, J & K 190006 Srinagar, India; 2 Department of Bio-Resources, Government Degree College for Women, Pulwama, J & K India; 3 Department of Zoology, University of Kashmir, Srinagar, J & K 190006 India; 4 Department of Pharmaceutics College of Pharmacy, King Khalid University, Abha, Saudi Arabia; 5 Department of Pathology College of Medicine, King Khalid University, Abha, Saudi Arabia; 6 Department of Biochemistry, University of Kashmir, Srinagar, J & K 190006 India

**Keywords:** Naringenin, Breast cancer, Network pharmacology, Molecular modelling, MCF-7 cells, Cancer, Breast cancer

## Abstract

Naringenin (NAR), a flavanone abundant in citrus fruits, has shown antiproliferative effects in several cancers, including breast cancer. However, its precise molecular mechanisms remain unclear. This study integrates network pharmacology, molecular modeling, and in vitro assays to investigate the anti-breast cancer potential of NAR. Target Genes associated with both NAR and breast cancer were identified through multiple databases, yielding 62 overlapping genes, which were further analyzed via a protein–protein interaction (PPI) network. Gene Ontology (GO) and KEGG pathway enrichment analyses revealed key involvement of PI3K-Akt and MAPK signaling pathways in NAR’s mechanism of action. Molecular docking studies showed strong binding affinities of NAR with key targets SRC, PIK3CA, BCL2, and ESR1, findings supported by molecular dynamics (MD) simulations, which confirmed stable protein–ligand interactions. Cell-based assays using MCF-7 human breast cancer cells demonstrated that NAR inhibits proliferation, induces apoptosis, reduces migration, and increases reactive oxygen species (ROS) generation. These results validate computational predictions and suggest that SRC may be a primary target mediating NAR’s anticancer activity. Collectively, this study provides mechanistic insights into the anti-breast cancer action of NAR and supports its potential as a lead compound for the development of SRC-targeted breast cancer therapies.

## Introduction

Cancer is a worldwide problem, with GLOBOCAN 2020 data reporting over 19.3 million new cases, and approximately 10 million mortalities in 2020 due to this deadly disease. Notably, female breast cancer overtook lung cancer, emerging as the most frequently occurring cancer (constituting 11.7% of the total cases), followed by lung cancer (11.4%), colorectal cancer (10%), and prostate cancer (7.3%)^1^^[–3^. Radiation therapy, mammography, chemotherapy, hormone therapy and surgery are the most frequently employed therapeutic approaches in the diagnosis and management of breast cancer^4–7^ Multidrug resistance (MDR) presently is a profound concern inherent in the existing therapeutic approach as due to this, the survival rate of individuals diagnosed with breast cancer has significantly declined^8^.

Therefore, novel therapeutic approaches that can inhibit metastasis and cellular migration and enhance clinical outcomes in breast cancer patients are extremely important^9^. Recent years have seen an upward trend in the integration of computational systems biology with experimental approaches. This has opened up new opportunities for drug discovery^10,11^ however, despite these advances, our understanding of the underlying mechanisms of natural medicines remains limited, and comprehending the intricate interactions among their complex components is still challenging.

For decades, natural products have played a vital role in traditional and folk medicine-based cancer therapies^12,13^. Among them, plant-derived secondary metabolites, particularly polyphenolic compounds such as flavonoids, have attracted significant attention due to their potential therapeutic properties^14,15^. Numerous studies highlight the anti-cancer effects of flavonoids, emphasizing their role as anti-carcinogens that inhibit cancer cell proliferation^15,16^. Various flavonoids have been isolated from plants and evaluated to understand their mechanisms of action against cancer. Recent research indicates that flavonoids function as antioxidants, regulating reactive oxygen species (ROS) production. This regulation promotes apoptosis in cancer cells while protecting normal cells from oxidative DNA damage^17,18^.

Among the various potent natural anticancer molecules, Naringenin (NAR), chemically known as 5,7-dihydroxy-2-(4-hydroxyphenyl)−2,3-dihydrochromen-4-one, is a hydrophobic citrus flavanone (a subclass of flavonoids) that belongs to the Vitamin P family^19–22^. Both in-vitro and in-vivo studies suggest that NAR exhibits potent antioxidant and anti-inflammatory properties, making it a potential therapeutic agent for conditions such as hepatitis, lung injury, diabetes, atherosclerosis, obesity, and cancer, with minimal systemic toxicity^23,24^. Preclinical studies indicate that NAR may be effective against several types of cancer, including prostate, breast, pancreatic, gastric, and lung cancer^25,26^. The therapeutic effects of NAR are primarily attributed to its capacity to modulate critical signaling pathways—including AKT, ERK, RTK receptors, NF-κB, TGF-β1, and MAPK—leading to the induction of apoptosis and suppression of cancer cell proliferation^27–30^. Additionally, in certain cancers, such as breast cancer, NAR has been found to reverse multidrug resistance in cancer cells^31,32^. While its efficacy in suppressing tumor growth and metastasis is well-documented, the precise mechanisms underlying its action remain largely unexplored.

Network pharmacology is an emerging interdisciplinary field that combines physiology, computational systems biology, and pharmacology^33^. In recent years, it has been widely used in understanding the pharmacological mechanism of drugs and drug discovery^34^.

With the aim to forecast the underlying mechanism of action of NAR and identify potentially effective components against breast cancer, we screened breast cancer target genes according to the disease database and identified key components and intersection genes by constructing drug-target network. Core targets with relatively high interaction relationships were predicted based on the degree values.

Using GO (gene ontology) analysis and KEGG (Kyoto encyclopaedia of genes and genomes), we performed further enrichment analysis on the intersection targets to predict the biological functions of the intersection genes and the main signal pathways of enrichment. For verification of the reliability of the selected key compound and core targets, we used molecular docking and Molecular dynamics (MD) simulation analysis to simulate the binding affinity between NAR and the core targets. Furthermore, in-vitro experiments were conducted to explore the naringenin’s effects on breast cancer.

## Network pharmacology analysis

### Screening of targets of NAR and breast cancer

NAR was screened for the target proteins using databases like SwissTargetPrediction (STP) (https://pubmed.ncbi.nlm.nih.gov/31106366/)^35,36^ and STITCH (https://pubmed.ncbi.nlm.nih.gov/18084021/)^37^. The canonical SMILES of the compound, with species specified as Homo sapiens were given as inputs. The protein targets were further screened from these databases with the criteria of probability value > 0.1 for STP and a score ≥ 0.8 for STITCH^37–39^. A STITCH score ≥ 0.8 indicates strong, high-confidence links between chemicals and proteins, based on experimental data, databases, and text mining. An STP probability > 0.1 helps identify likely targets without excluding too many possibilities^40,41^. The protein targets associated with breast cancer were obtained from databases as OMIM (Online Mendelian Inheritance in Man, **(**https://www.omim.org/)^42^, CTD (Comparative Toxicogenomics Database, (https://ctdbase.org/)^43^ and GeneCards (http:/www.genecards.org/)^44^ using “Breast Cancer” as the keyword and based on the GIFT (GeneCards Inferred Functionality) score of > 50, the targets were screened^38,45^. Subsequently, the targets obtained from individual databases were consolidated, and all duplicate entries were eliminated.

### Druggability screening and identification of common targets

To evaluate the druggability of protein targets associated with NAR and breast cancer, the online druggability prediction tool named Drugnome AI (http:/astrazeneca-cgr-publications.github.io/DrugnomeAI/index.html)^46^ was utilized. Protein targets with raw Targets possessing raw druggability scores ≥ 0.5 were considered potentially druggable. Subsequently, common targets between NAR and breast cancer were identified using the online tool Venny v2.0.2 tool (https://bioinfogp.cnb.csic.es/tools/venny/index2.0.2.html).

### Construction of protein-protein interaction (PPI) network

Protein-protein interaction data for shared targets between NAR and breast cancer were retrieved from the STRING (http://string-db.org/) database using a high-confidence score threshold (≥ 0.7)^42^. The interaction data derived from STRING database was illustrated into a network graph using Cytoscape software v3.9.1. Subsequently, topological analysis of the network was performed using the CytoNCA plug-in to calculate closeness centrality (CC), eigenvector centrality (EC), betweenness centrality (BC), and degree centrality (DC). Nodes exhibiting CC, EC, BC, and DC values above the average were subjected to a double-screening process to identify the key targets.

### Gene ontology (GO) and Kyoto encyclopedia of genes and genomes (KEGG) pathway analysis

Gene enrichment and metabolic-pathway analysis of the common targets were conducted using the ShinyGO v0.80 online server (http://bioinformatics.sdstate.edu.go)^47^. For the analysis, the selected species “Homo sapiens,” and the false discovery rate (FDR) cut off was set to 0.05. The results of the enrichment analysis, specifically the Gene ontology (GO) terms categorized into biological process (BP), cellular components (CC), and molecular functions (MF), were visually represented as bubble charts.

## Expression analysis

### TIMER 2.0

TIMER 2.0 (http://timer.cistrome.org/) is a comprehensive internet-based tool for analyzing gene expression patterns and immune cell infiltration across multiple cancer types using TCGA data^37,38^. It facilitates the exploration of relationships between Gene expression, mutation status, and immune cell infiltration through various algorithms such as CIBERSORT, quanTIseq, MCP-counter, and EPIC. In this study, TIMER 2.0 was employed to analyze the expression pattern of SRC across different cancer types. The platform provides a systematic approach to estimating immune infiltrates and evaluating gene expression across tumors (http://timer.comp-genomics.org/).

### UALCAN

UALCAN^48^ is an interactive web portal that facilitates the analysis and visualization of cancer transcriptome, proteomics, and patient survival data. It leverages datasets from The Cancer Genome Atlas (TCGA) to enable users to explore the expression of protein-coding Genes and evaluate their prognostic significance across 33 different cancer types. This tool is widely used in cancer research for assessing gene expression patterns, clinical correlations, and survival analyses.

### bc-Gen exminer

The Breast Cancer Gene-Expression Miner (bc-Gen EXminer 4.5) web source (http://bcgenex.ico.unicancer.fr)^49^ is a user-friendly, web-based application designed to assess the prognostic significance of genes in breast cancer through its prognostic module. This tool was utilized to explore the relationship between *SRC* and key pathophysiological characteristics in breast cancer patients. Additionally, bc-Gen EXminer 4.5 was employed to analyze the correlation between *SRC* expression levels and various clinical parameters in breast tumours, including HER2 enrichment, p53 mutation status, hormonal status (estrogen & progesterone), and Scarff-Bloom-Richardson (SBR) grade.

### GEPIA2: (Gene expression profiling interactive analysis)

GEPIA, (http://gepia.cancer-pku.cn/)^50^ is a web-based tool used to deliver fast and customizable functionalities based on TCGA and Genotype-Tissue Expression (GTEx) data. GEPIA provides key interactive and customizable functions including differential expression analysis, profiling plotting, correlation analysis, patient survival analysis, similar gene detection and dimensionality reduction analysis. With an intuitive interface and simple click-through options, GEPIA enables rapid exploration of differential gene expression, survival analysis, and correlation studies across multiple cancer types and normal tissues. By bridging the gap between large-scale cancer genomics data and user-friendly visualization, GEPIA plays a pivotal role in advancing scientific discovery, fostering data-driven discussions, and accelerating therapeutic research.

### Kaplan–Meier plotter

The Kaplan–Meier plotter is a bioinformatic tool that integrates gene expression profiles with survival data from cancer patients. This online tool was used to assess the prognostic value of *SRC* in breast cancer patients. To analyze overall survival (OS) and recurrence-free survival (RFS), patient samples were categorized into high- and low-expression groups based on the median gene expression level. The hazard ratios (HR), along with their 95% confidence intervals (CI) and log-rank p-values, were calculated to determine statistical significance, with *p* < 0.05 considered as the threshold for significance.

## Molecular modelling studies

### Molecular docking analysis

Molecular docking studies were carried out between the target proteins (*SRC*, *PIK3CA*, *BCL2* and *ESR1*) with NAR using Autodock version v 4.2.1^51,52^. The Research Collaboratory for Structural Bioinformatics-protein data bank (RCSB- PDB) was utilized for extraction of crystal structures of all the target proteins and experiments were performed in triplicates. In order to obtain the best cluster having lowest energy score with high number of populations, RMSD clustering maps were obtained by re-clustering with a clustering tolerance of 0.5 Å, 1 Å, and 2 Å respectively. The ligand binding sites were determined on the basis of literature survey for *SRC*, *PIK3CA*, *BCL2* and *ESR1*.

### Molecular dynamics simulation (MDS) analysis

The molecular dynamics simulations were carried out for *SRC* and NAR complex using the tool Desmond 2020.1 from Schrödinger, LLC and independent simulations were carried out at 37℃. Explicit solvent model with SPC water molecules and the OPLS-2005 force field in a period boundary salvation box with dimensions of 10 Å x 10 Å x 10 Å were used in this system^53–56^. Sodium (Na^+^) ions and 0.15 M sodium chloride (NaCl) solution was utilized to neutralize the charge and simulate the physiological environment to the system. Initially, the system was equilibrated using the Conical (NVT) ensemble for 10 ns to retrain over the protein ligand complexes. An isothermal-isobaric (NPT) ensemble was used for a brief run of equilibration and minimization lasting 12 ns after the preceding phase. The NPT ensemble was set up using the Nose-Hoover chain coupling scheme and throughout the simulation process, temperature, relaxing time of 1.0 ps, and 1 bar pressure was maintained^57^. A time step of 2 fs was used. To maintain the pressure control with a relaxation time of 2 ps, Martyana-Tuckerman-Klein coupling scheme barostat method was adopted^58^. Long-range electrostatic interactions were calculated using the particle mesh Ewald approach^59^and the radius for the coulomb interactions was set at 9Å. For each trajectory, bonded forces were calculated using RESPA integrator for time step of 2 fs. Final production run was carried out for 100 ns each. A 100 ns simulation was chosen to ensure adequate sampling of the protein-ligand complex’s dynamic behaviour, providing enough time to observe stable binding interactions and relevant conformational changes without excessive computational cost. The 100 ns simulation has been earlier used by^60,61^. For stability monitoring in MD simulations, following tools were calculated; root mean square deviation (RMSD), radius of gyration (Rg), solvent accessible surface area (SASA), and root mean square fluctuation (RMSF)^62^.

### Principal component (PCA) analysis

Geo_measures v 0.8 was used to measure the free energy landscape (FEL) based on principal component analysis (PCA) complexes^63^. Geo_measures v 0.8 include a library of g_sham and eigen values recorded in a 3D plot using the matplotlib python package.

## Experimental analysis

### Cell line and culture

MCF-7 cells (ATCC) cells (passage number 2) were cultured in DMEM medium which was supplemented with 10% FBS (fetal bovine serum and supplied with 1% PEN-STREP (penicillin-streptomycin) antibiotic and maintained at 5% carbon dioxide incubator at 37 °C. Cells were routinely tested for mycoplasma contamination using PCR-based assay.

### Antiproliferative assay

To determine the antiproliferative effect NAR, 3-(4,5-dimethylthiazol-2-yl)−2,5-diphenyltetrazolium bromide (MTT) assay was performed in accordance with the method followed Rui Wang et al. with slight modifications^64^. MCF-7 cells (10^4^ cells/100 µL were seeded at an initial density of 1 × 10^4^ cells/100 µL in 96 well plate for 24 h. Cells were treated with different concentrations (10, 20, 40, 80, 160, & 320 µM) of the drug with vehicle control dimethyl sulfoxide (DMSO) for 72 h. After the treatment 10 µL of MTT (5 mg/mL in PBS) was added for 4 hours in CO_2_ incubator. The supernatant from each well was carefully removed and 100 µL of DMSO added to solubilize the formazan crystals. The final absorbance was measured at 570 nm using microplate reader (Bio-Rad USA) and IC_50_ was determined.

### Wound healing assay

Proliferating MCF-7 cells were seeded and allowed to grow in 6 well plate in a density of 1.5 × 10^6^ cells/well until a monolayer was formed. The assay was performed according the Xiaojuan Wang et al.’s method with slight modifications^65^. After the monolayer formation a wound was given to the cell by holding the 200 µL pipette tip vertically. Detached cells were removed by 1X PBS washing and treatment of NAR with different concentrations (50, 100, and 150µM) was given. The scratch closure was monitored and Images were taken at different intervals of time with Nikon Eclipse Ti, USA Andor Clara DR-4285 TI microscope at 10x magnification after the treatment. To study cell migration, closure of the wound was measured using Image J 1.54 g software version Java 1.8.0_345 (64 bit) https://imagej.net/ij/download.html.

### Cell viability by PI staining

For this, a slightly modified version of Hongyun Cheng et al.‘s PI Staining method was employed^66^. MCF-7 cells were seeded at a density of 1.5 × 106 cells/well in a 6-well plate, cells were exposed to 50, 100, and 150 µM of NAR for 24 h. After treatment cells were harvested by trypsinization and then centrifuged for five minutes at 1000 rpm. Following this, cells were given 1X PBS washing and centrifuged again at 1500 rpm for 5 min. After this each to FACS tube 1 µl of PI was added and kept in dark for 15 min, and analyzed by flowcytometry for cell viability.

### ROS analysis by flowcytometry

The assessment of reactive oxygen species (ROS) was conducted following a previously established protocol by Ming Zhang et al., with minor adjustments^67^. After exposure to varying concentrations of NAR (50 and 100 µM) for 24 h, cells were detached using trypsinization. The collected cells were then centrifuged at 1000 rpm for 5 min, and the resulting pellet was resuspended in 200 µl of 0.5 µM 2՛,7-dichlorodihydrofluorescein diacetate (DCFDA) dye (5 mM in DMSO). The samples were incubated at 37 °C for 25 min, protected from light using aluminium foil. ROS levels were quantified based on median fluorescence intensity (MFI) through flow cytometry using the Attune NxT acoustic focusing cytometer (USA) and analyzed with FlowJo VX version 10.1 software. The stock solution of DCFDA exhibited excitation and emission wavelengths of 495 nm and 529 nm, respectively.

### Statistical analysis

One way ANOVA was used for the analysis using Graph Pad Prism V8.0.2 software. Data is expressed as as Mean ± SD. All the experiments were carried out in triplicates to ensure the reproducibility.

## Results

### Network pharmacology analysis

#### Target collection and PPI network

**Fig. 1:**
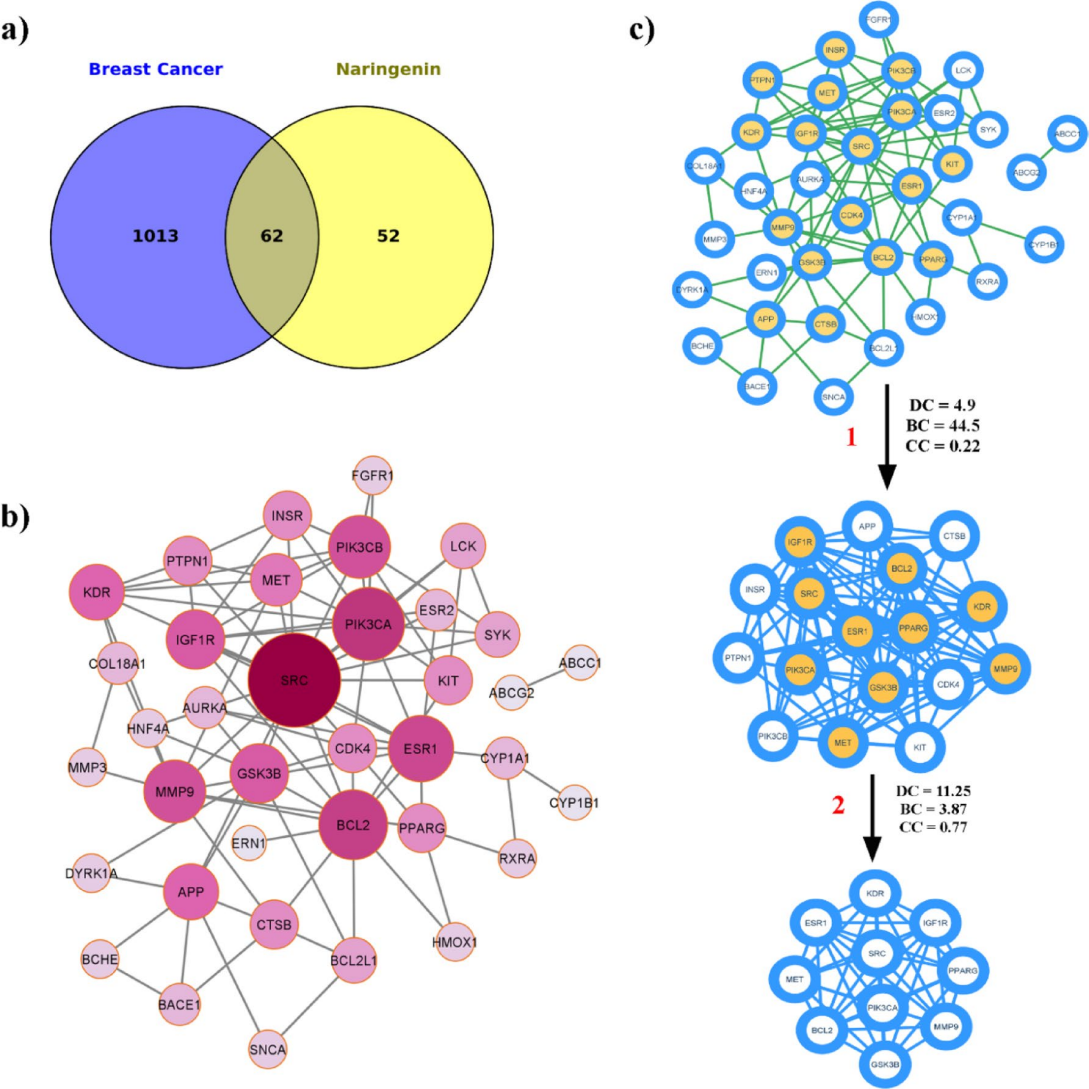
Protein-protein interaction network. (**a**) Venn diagram illustrating the overlap of gene targets between breast cancer and naringenin. (**b**) Protein-protein interaction (PPI) network of common genes, sorted by degree. (**c**) Refinement of the PPI network using topological parameters of the network.

In this study, a total of 1,075 Gene targets related to breast cancer and 114 targets associated with NAR were collected from various databases. Among them, 62 targets were found to be common to both categories, with 43 of these predicted to possess a druggability score exceeding 0.5. A Venn diagram (Fig. 1a) was then created to illustrate the overlap between breast cancer and NAR targets. Subsequently, a protein-protein interaction (PPI) network for the common druggable targets was constructed using the String database. The resulting network (Fig. 1b) consisted of 43 nodes and 99 edges, with nodes sorted by degree. Utilizing the CytoNCA package in Cytoscape software, core targets were identified from the PPI network (Fig. 1c). Initial screening of nodes was based on topological parameters such as Degree centrality (DC), Betweenness centrality (BC), and Closeness centrality (CC). The average values of DC, BC, and CC were found to be 4.918, 44.540, and 0.223 respectively. Nodes surpassing these average values were selected for further network construction, resulting in a secondary PPI network with 17 nodes and 58 edges. The average values of DC, BC, and CC for this network were 11.25, 3.87, and 0.774 respectively. Further refinement of nodes with average topological parameter values led to the construction of a final PPI network consisting of 10 nodes and 42 edges. Details of DC, BC, and CC values are provided in Table 1. Remarkably, among the nodes in the final PPI network, *SRC*, *PIK3CA*, *BCL2*,* and ESR1* exhibited the highest DC, BC, and CC. This suggests that the NAR could potentially provide therapeutic benefits against breast cancer by targeting *SRC*, *PIK3CA*, *BCL2*,* and ESR1.*

**Table 1:** The topological parameters of the twice-screened PPI network.

Gene	Degree Centrality	Betweenness Centrality	Closeness Centrality
*SRC*	16	12.81	1
*PIK3CA*	15	7.67	0.94
*BCL2*	14	5.86	0.89
*ESR1*	14	7.54	0.89
*PIK3CB*	14	8.78	0.89
*MMP9*	14	9.9	0.89
*IGF1R*	12	2.76	0.8
*GSK3B*	12	3.56	0.8
PPARG	12	4.35	0.8
MET	12	4.5	0.8

#### Gene ontology and KEGG pathway analysis of breast cancer targets and NAR

**Fig. 2:**
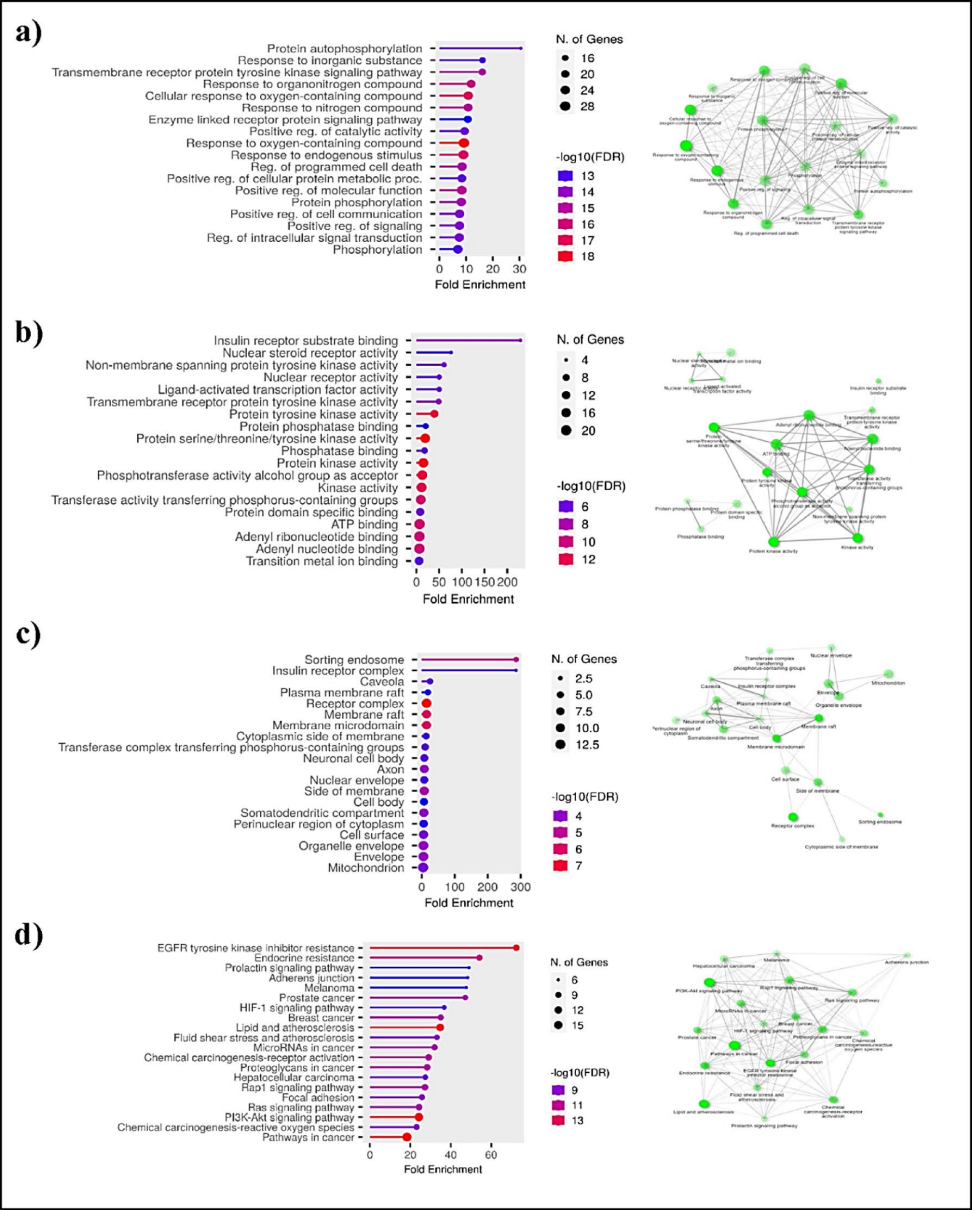
Gene Ontology and KEGG pathway analysis of the breast cancer targets of naringenin. (**a**) BP, (**b**) MF, (**c**) CC and (**d**) KEGG pathways.

Figure 2 illustrates the Gene ontology enrichment analysis for breast cancer targets of the NAR. A total of 1505 Gene Ontology (GO) terms were associated with the identified breast cancer targets. These GO terms were categorized into biological processes (BP), cellular components (CC), and molecular functions (MF). Specifically, 1000 GO terms were enriched under BP, 193 under CC, and 312 under MF for breast cancer targets of NAR. Within the BP category, response to oxygen-containing compounds, cellular response to oxygen containing cpds terms were predominated, reflecting the significance of response to oxygen containing cpds in breast cancer, where dysregulation is commonly observed^68,69^. It has been documented that breast cancers and the surrounding tumor microenvironment (TME) cells have higher levels of reactive oxygen species (ROS). Because they facilitate reciprocal communication between different elements and play an important role in the development and spread of tumors^70^. ROS are critical factors in breast TME as they ensure bidirectional communication among various components and mediate multi-faceted roles in tumor progression and metastasis. Other enriched BP terms include those associated with protein autophosphorylation, cell growth regulation, signal transduction, and cell death^69^. A network was constructed using highly enriched BP GO terms as nodes, with the number of associated genes determining node degree (Fig. 2a). This network comprised 18 nodes and 146 edges, with master nodes such as response to oxygen-containing compounds, cellular response to oxygen containing compounds and response to endogenous stimulus showing high connectivity.

Figure 2b depicts a bar plot illustrating enriched GO terms in the MF category. Highly enriched MF GO terms included kinase activity, nucleotide binding, and phosphotransferase activity. The network revealed significant connectivity among terms associated with kinase activity, highlighting the pivotal role of kinase enzymes in cell-to-cell communication, transduction of extracellular signals to pathways related to cell growth, cell proliferation and migration in breast cancer. Many studies have shown that there is spectrum of protein kinases which are highly expressed in human breast cancer cells. Dysregulation of kinase activity encompassing expression levels, aberrant phosphorylation, and mutations, is closely linked to breast cancer^71^. In terms of CC, enriched GO terms were related to various cellular components such as caveola, plasma membrane raft, insulin receptor complex and mitochondria (Fig. 2c). A network was constructed based on highly enriched CC GO terms contained 20 nodes and 61 edges, with membrane-based terms like membrane raft and membrane microdomain exhibiting notable connectivity and there were many overlapped genes shared between membrane raft and membrane microdomain.

In recent studies, it has been verified that membrane lipid rafts (which represent a sort of lipid-based structures that regulate the assembly and functioning of numerous cell signalling, tumor cell growth, adhesion, migration, invasion and apoptosis) which are related to cancer are highly found in cancerous cells than normal cells leading to tumor progression and formation of membrane raft microdomain, involved in triggering specific events during apoptosis execution, i.e., mitochondria hyperpolarization and depolarization, with consequent release of apoptogenic factors^72,73^. Notably, recent studies have focused on developing drugs which can modify the lipid composition plasma membrane of cancer cells, aiming to enhance the uptake of cancer therapeutics^73,74^.

**Fig. 3:**
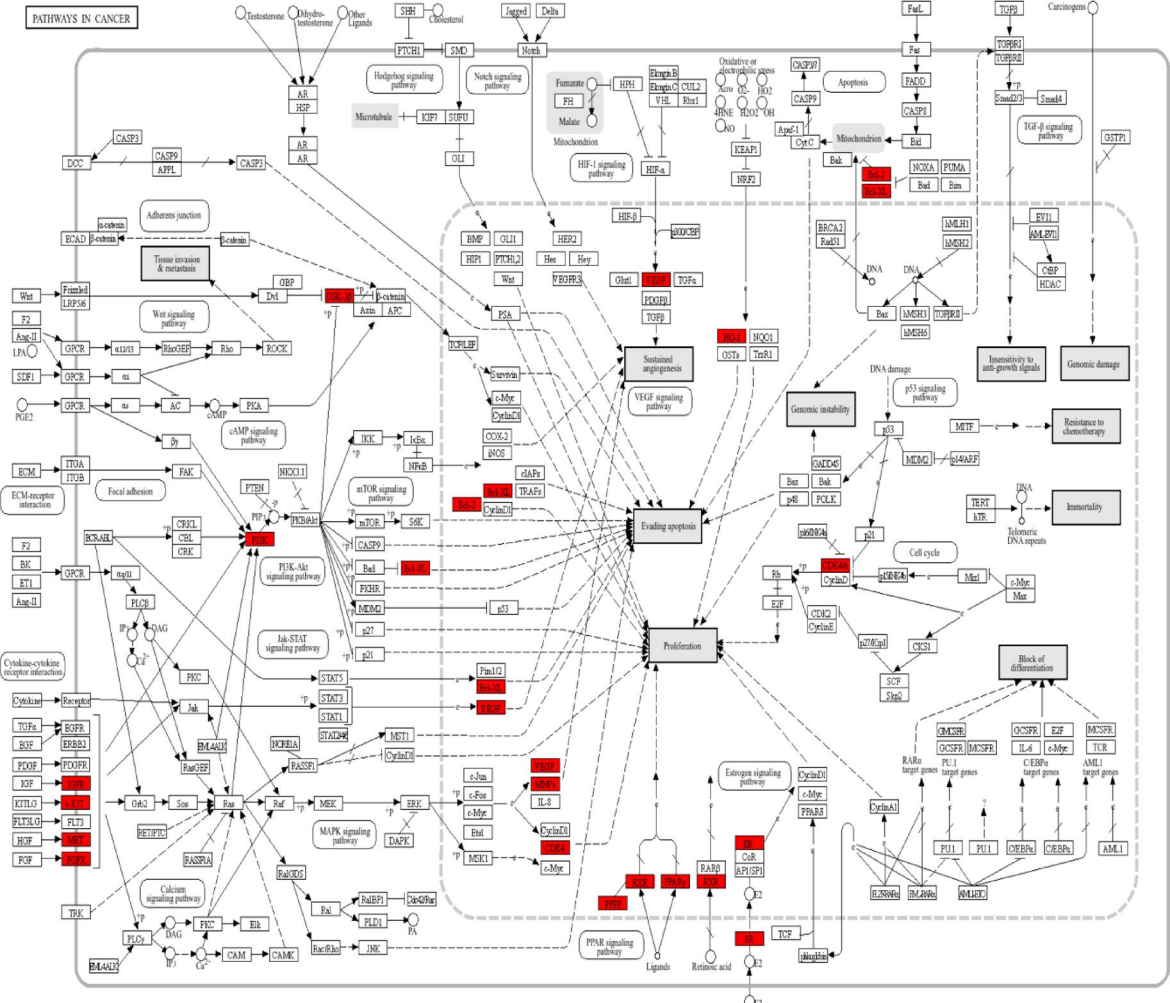
Breast cancer targets of naringenin associated with KEGG pathway “pathways in cancer”^75–77^.

Figure 2d presents the outcome of the KEGG pathway analysis. The pathways associated with common genes shared between breast cancer and the synthesized compounds are showcased, encompassing pathways in cancer, PI3K-Akt signalling, MAPK signalling, EGFR Tyrosine kinase inhibitor resistance and others (Fig. 3).

Notably, the majority of these enriched pathways exhibit a strong association with breast cancer. A network was constructed based on the highly enriched pathways, comprising 20 nodes and 146 edges, with PI3K-Akt signalling pathways demonstrating a high level of interconnectedness. The genes linked to the prominently enriched KEGG pathway, “Pathways in cancer,” are depicted in the pathway diagram (Fig. 3**)**. Predominantly, the selected genes are associated with receptor interaction pathways, including cytokine and cytokine receptor interaction pathways. Additionally, some genes are linked to cell proliferation, cell apoptosis, estrogen signalling pathways and some genes in PPAR signalling pathways. This comprehensive analysis sheds light on the intricate relationships and key molecular players within these pathways that may contribute to the understanding of breast cancer and the potential therapeutic impact of NAR on the disease.

### Expression analysis

#### SRC is overexpressed in breast cancer

**Fig. 4:**
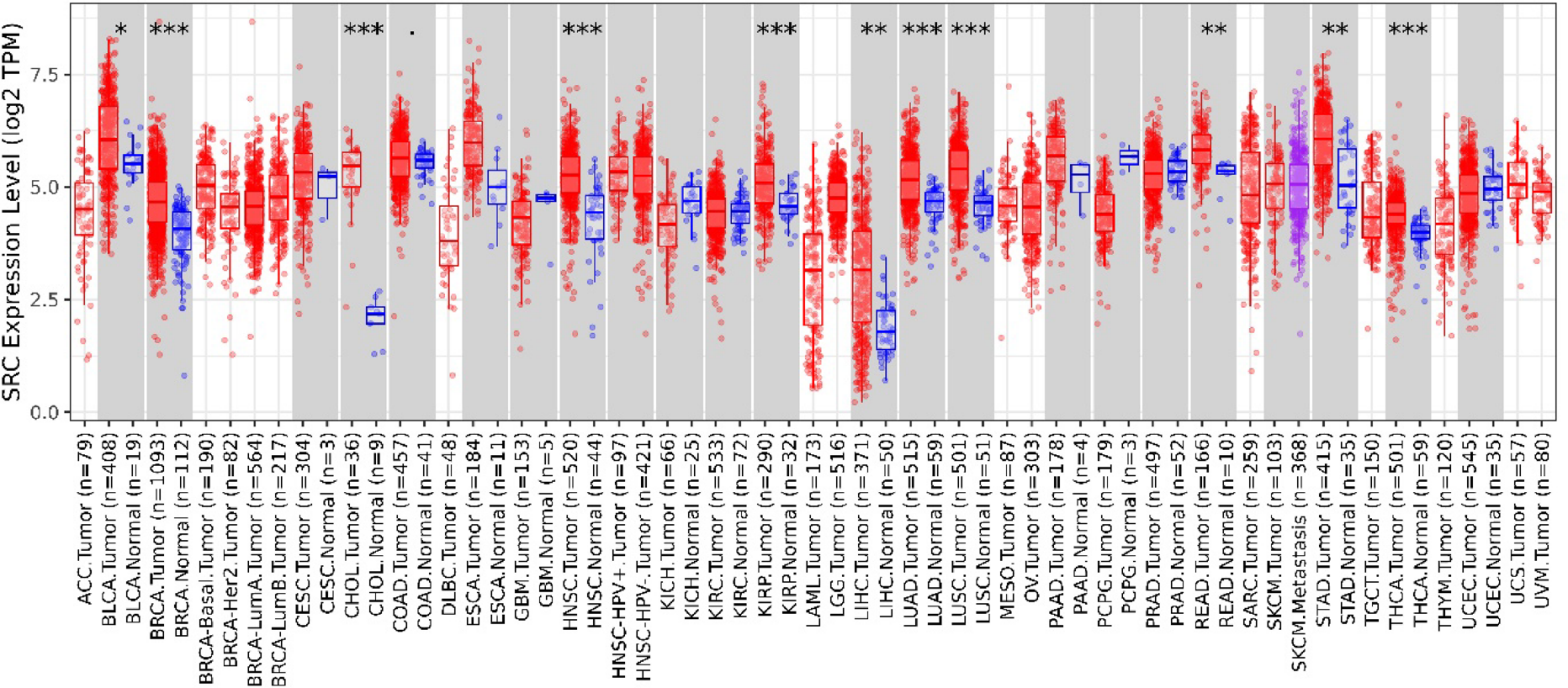
*SRC* is expressed differently in several malignancies compared to normal tissues. The *SRC* levels happen to be raised in numerous malignancies, including BC, as shown by the box plot.

**Fig. 5:**
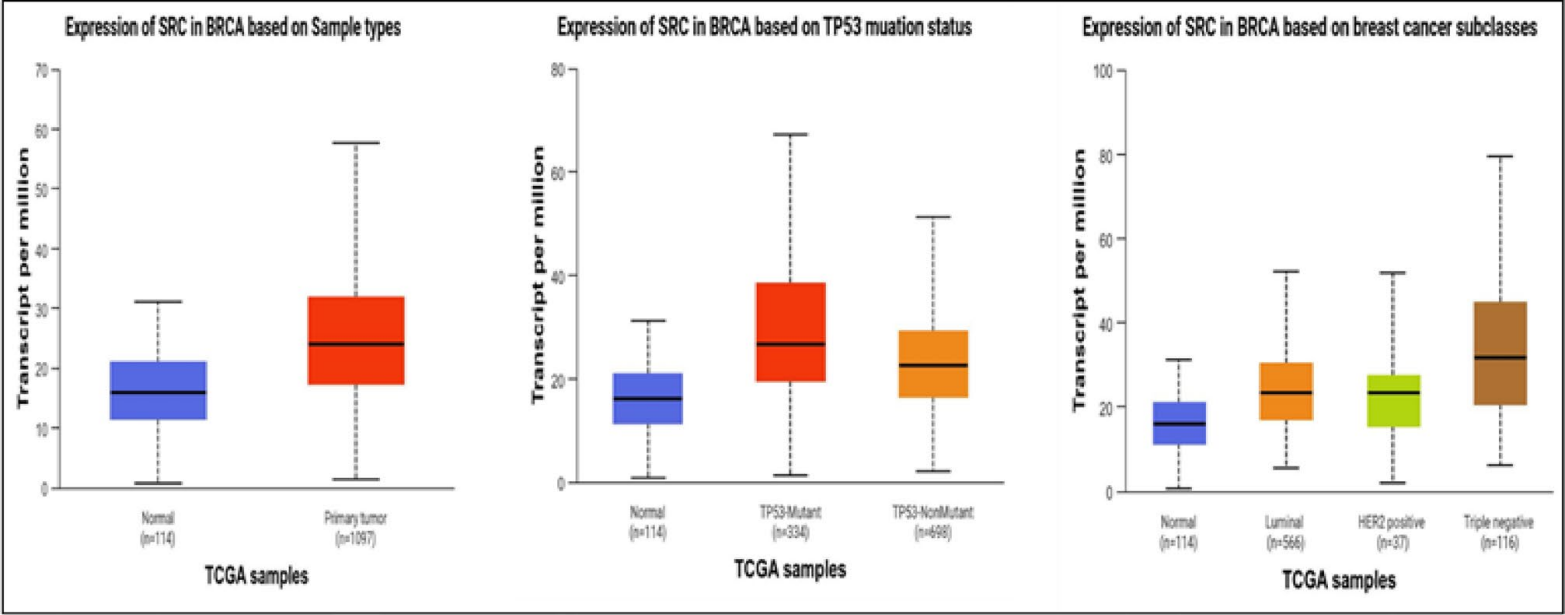
Expression pattern of *SRC* in major breast cancer subtypes using UALCAN (p value > 0.05).

Comprehensive analysis using the TIMER 2.0 database revealed that *SRC* is highly expressed across various malignancies, suggesting its critical role in tumorigenesis and disease progression (Fig. 4). Notably, its elevated expression in breast cancer (BC) indicates a potential oncogenic driver and therapeutic target, particularly in aggressive subtypes such as triple-negative breast cancer (TNBC). Further analysis using UALCAN confirmed that *SRC* expression is higher across different BC subtypes, especially TNBC. Additionally, a strong association was observed between *SRC* overexpression and p53 mutations, with *SRC* being upregulated across all tumor samples (Fig. 5).

**Fig. 6:**
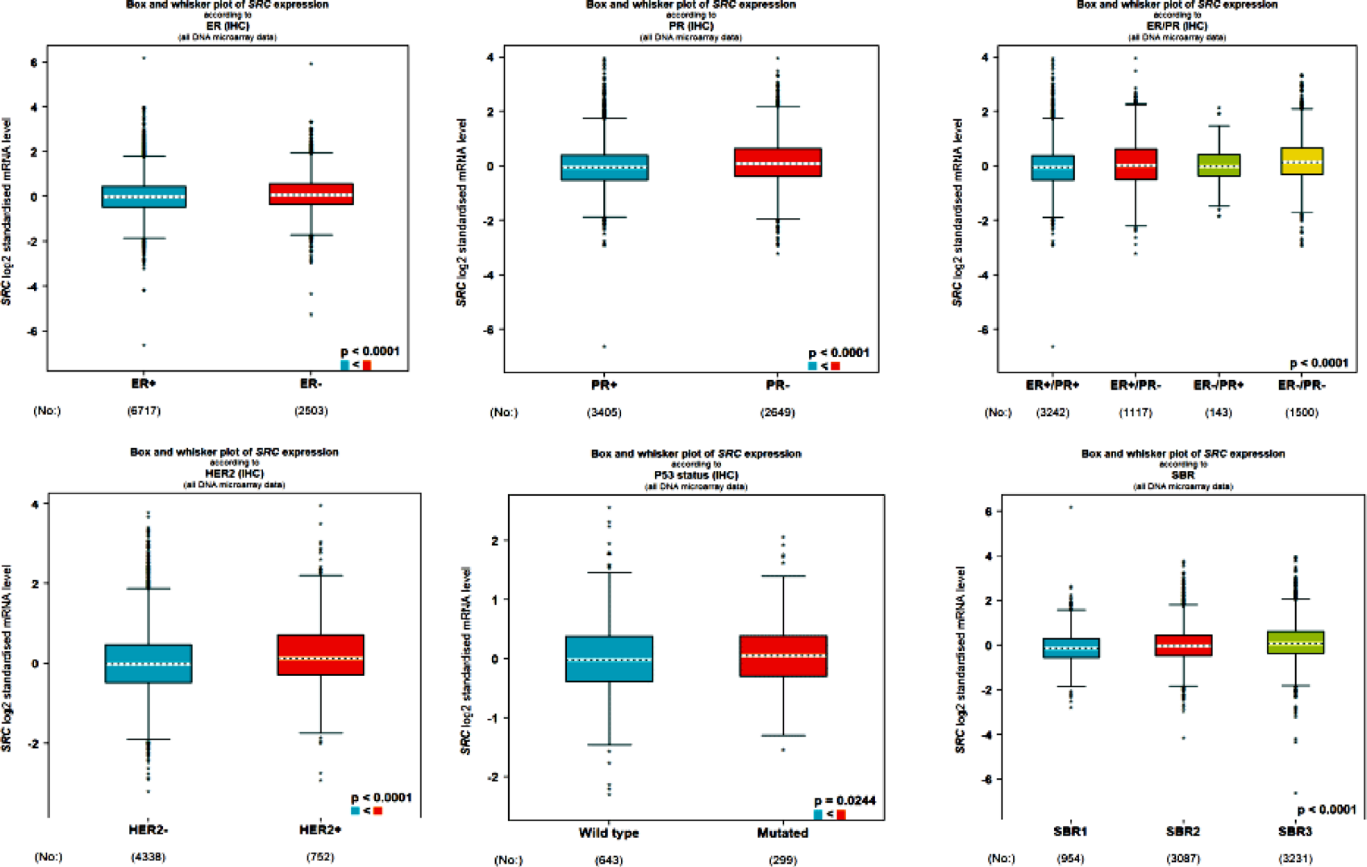
b-Gen EXminer analysis of *SRC*, showed a tie-up with clinicopathological parameters of BC i.e., Hormonal status, Her2 enrichment, SBR grade, and p53 status.

To further explore the relationship between *SRC* and key pathophysiological features in BC, the bc-Gen-ExMiner tool was employed. The results demonstrated significantly higher *SRC* expression in BC patients with hormone receptors (estrogen and progesterone) (*p* < 0.0001) and its association with SBR grade 1 and HER2 positivity (*p* < 0.0001). However, *SRC* expression did not exhibit significant variation among p53 subtypes (*p* = 0.0244) (Fig. 6).

**Fig. 7:**
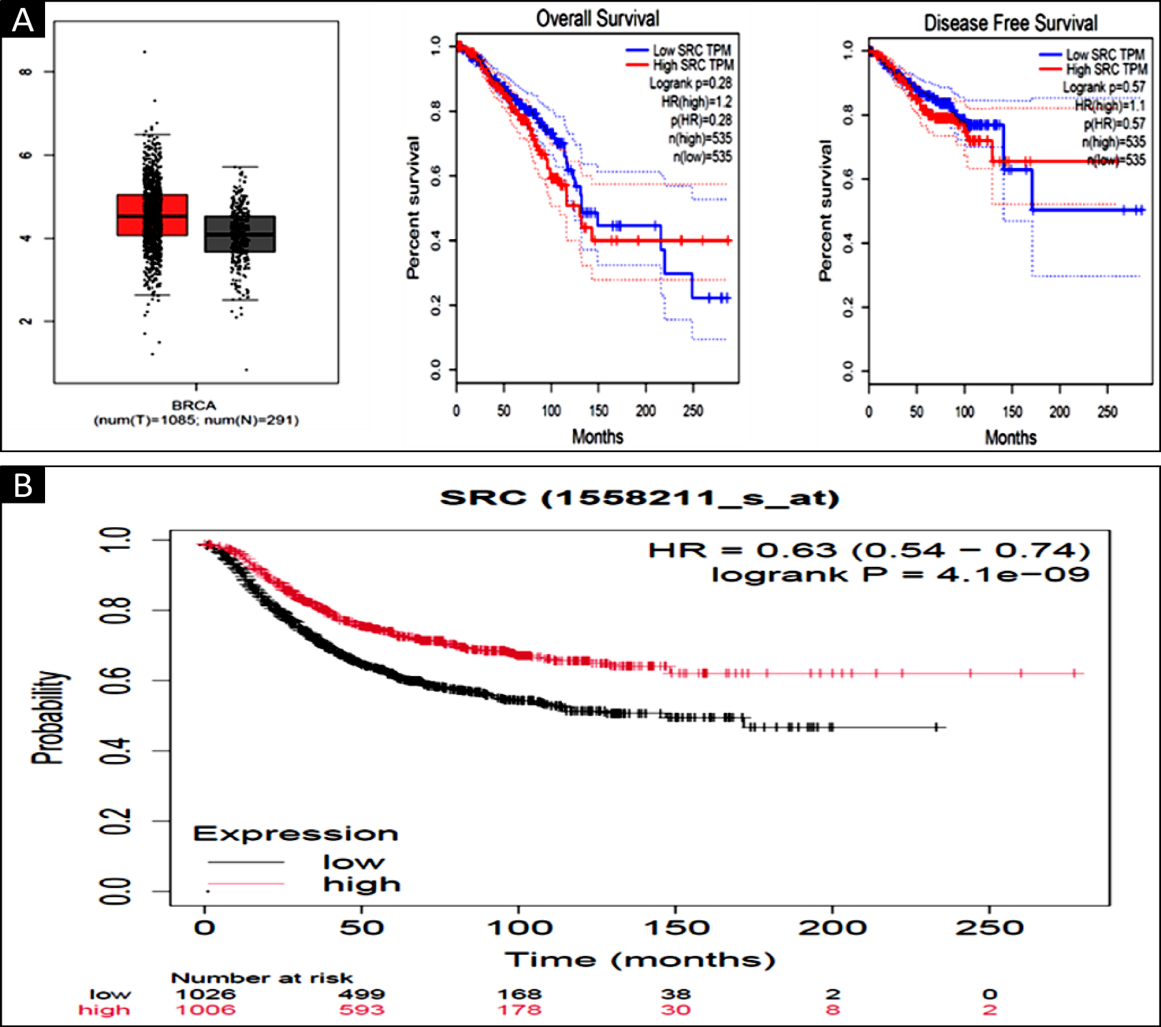
(**A**). Box plot, OS and DFS survival analysis and expression pattern of *SRC* analysed in BC by making use of Gepia2 web source (p value > 0.05). (**B**). Correspondence between expression of *SRC* with RFS in BC patients using Kaplan–Meier plotter.

The GEPIA web server, a widely used platform for gene expression analysis based on TCGA and GTEx datasets, further confirmed the elevated expression of *SRC* in BC patients. Importantly, patients with higher *SRC* expression exhibited poorer overall survival and disease-free survival (Fig. 7a). Additionally, survival analysis using the Kaplan–Meier plotter provided further evidence of the prognostic significance of *SRC*. Based on median expression levels, BC patients were categorized into high and low expression groups. A relapse-free survival (RFS) analysis conducted on 4,929 BC patients over a follow-up period of approximately 300 months revealed that higher *SRC* expression correlated with poorer RFS, with an HR of 0.63 and a highly significant p-value of 4.1e-09 (Fig. 7b).

## Molecular modelling studies

### Molecular docking studies of NAR in the active sites of SRC, PIK3CA, BCL2 and ESR1

**Fig. 8:**
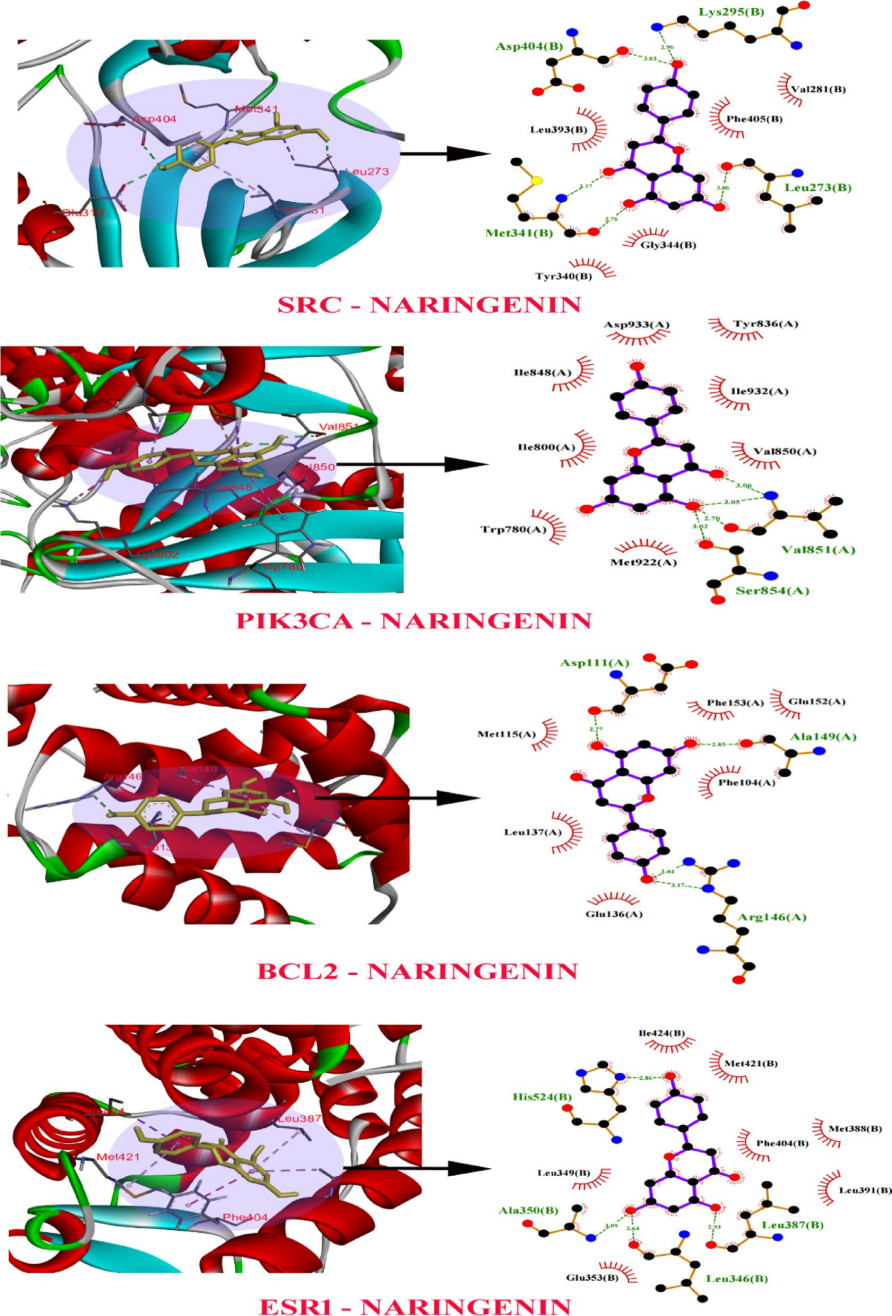

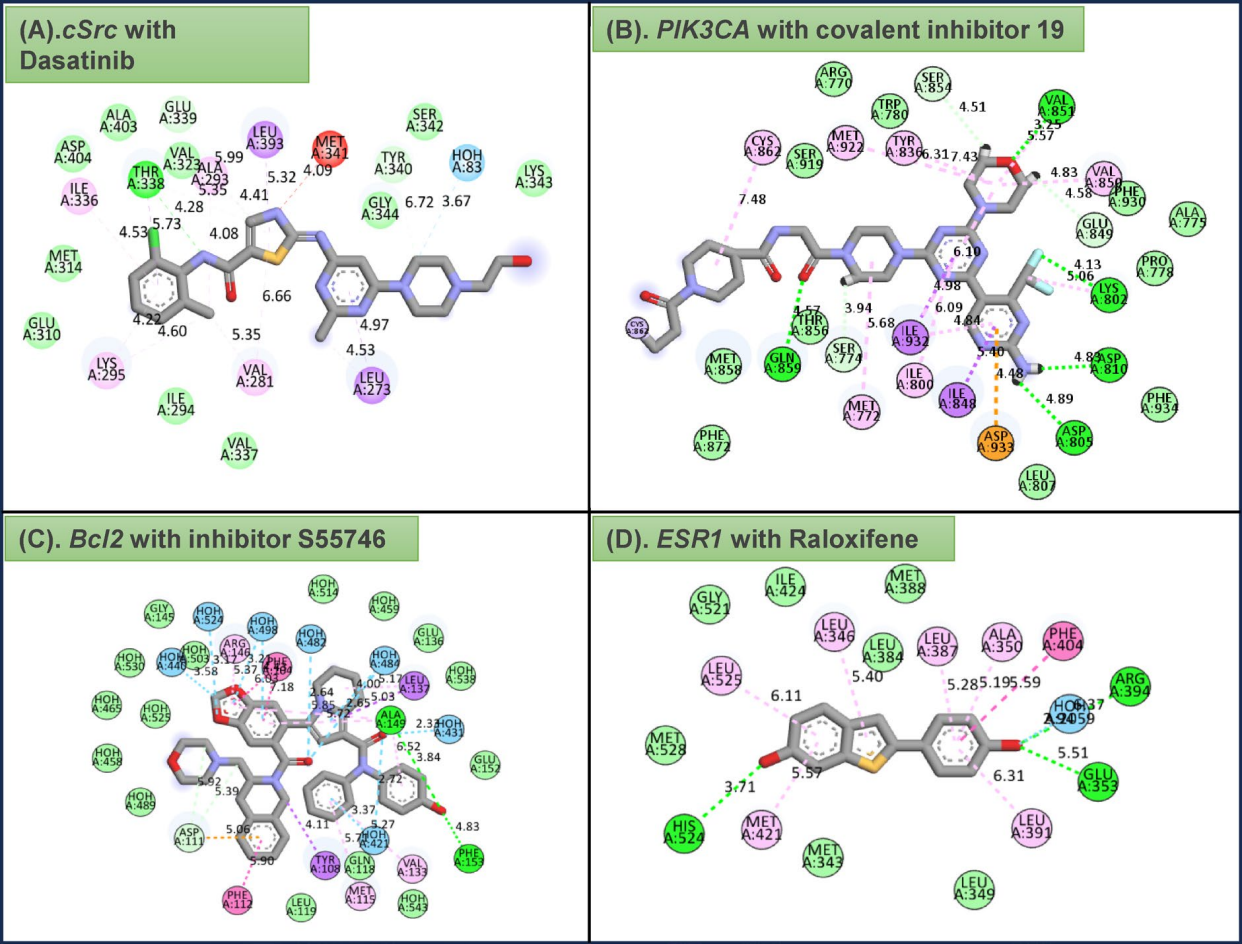
(**a**) 2D 3D interactions of compound NAR with different target proteins *SRC*, *PIK3CA*,* BCL2 and ESR1* active sites. (**b**) 2D interaction of standard drugs with (**A**) *SRC*, (**B**) *PIK3CA*, (**C**) *BCL2* and (**D**) *ESR1* target proteins active sites.

Molecular docking studies were performed to verify the affinity of target protein (s) and NAR. Based on the screened targets in the PPI network and the results of KEGG enrichment analysis, we identified *SRC* (PDB ID: 3F3V), *PIK3CA* (PDB ID: 7R9Y), *BCL2* (PDB ID: 6O0K) and *ESR1*(PDB ID: 1UOM) as core targets enriched in the breast cancer signalling pathway for molecular docking. Table 2 shows the minimum binding energy of the Ligand to the receptor after 50 simulated docking attempts. Figure 8a shows the optimal binding sites and hydrogen bonding positions of ligand and receptor.

**Table 2:** Binding energies (kcal/mol) obtained from the docking calculations of NAR with target proteins.

Compound name	Genes	Binding energy score	H-bonds
Naringenin	*SRC*	−9.2	Asp404, Lys295, Met341, Leu273
	*PIK3CA 7R9V*	−8.8	Val851, Ser854
	*BCL2 6GL8*	−7.1	Asp111, Ala149, Arg146
	*ESR1*	−8.6	His524, Ala350, Leu387, Leu346

NAR possessed the highest binding affinity amongst the hit compounds towards all the studied target proteins. 2D representations of the predicted binding mode for NAR with top breast cancer target proteins such as *SRC*, *PIK3CA*,* BCL2* and *ESR1* active sites are shown in Fig. 8b. NAR was strongly bound to the *SRC* protein, with the lowest binding energy of −9.2 kcal/mol. During the interaction of the ligand (NAR) with *SRC*, various amino acid residues such as ASP404, LYS295, MET341, LEU273 form the hydrogen bonding. Therefore, it can be inferred from the molecular docking study that NAR has a high affinity for *SRC* protein and should be considered for further MD simulation investigations.

### Molecular dynamic simulation analysis

#### RMSD

**Fig. 9:**
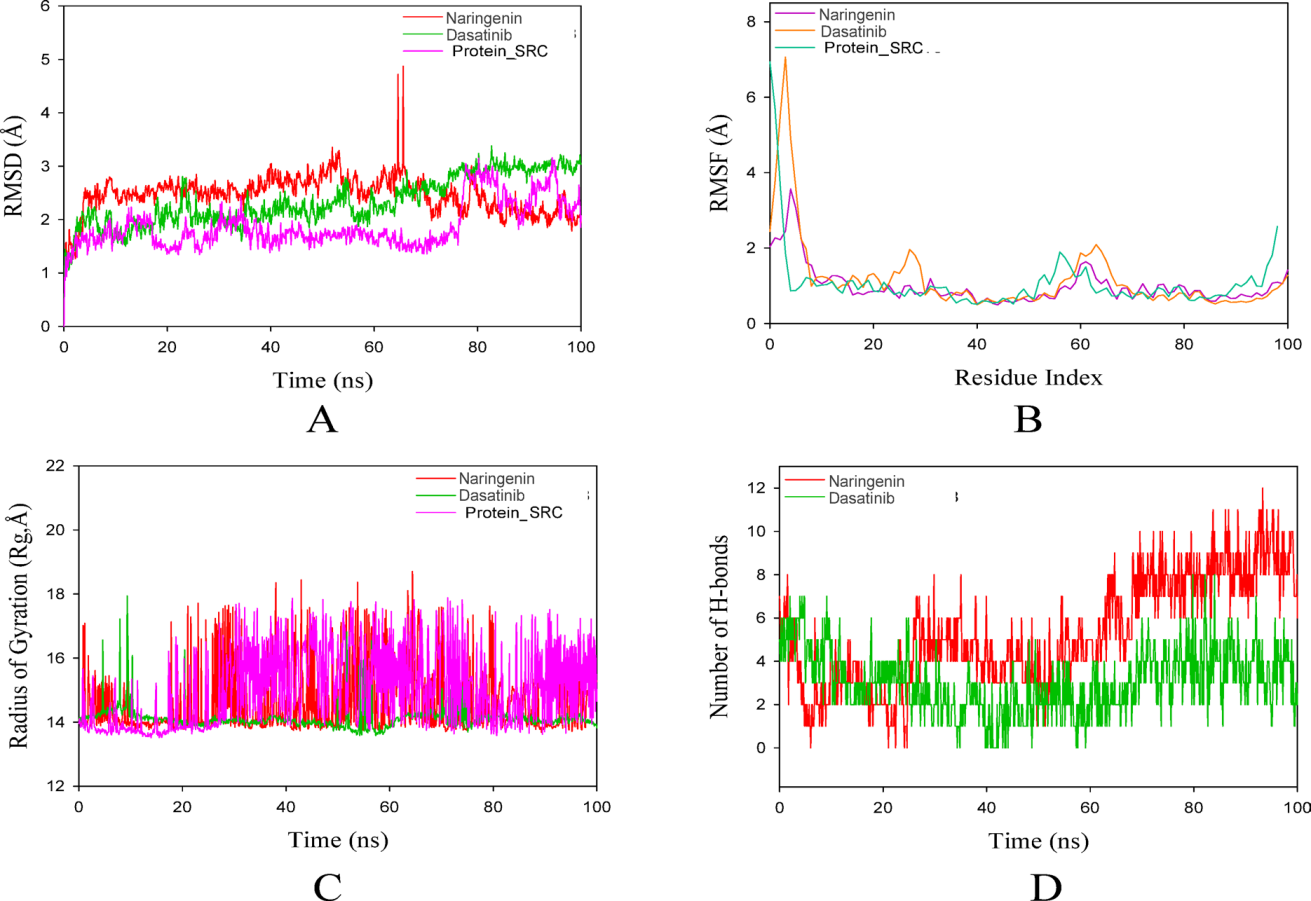
MD simulation analysis of 100 ns trajectories of (**A**). RMSD of the Cα backbone of *SRC*_NAR and *SRC*_Dasatinib, (**B**). RMSF of Cα backbone of *SRC*_NAR and *SRC* _Dasatinib, (**C**). The radius of gyration (Rg) of Cα backbone of *SRC*_NAR and *SRC* _Dasatinib, (**D**). The solvent-accessible surface area of the *SRC*_NAR and *SRC* _Dasatinib complex.

The root mean square deviation (RMSD) stands as the foremost and primary parameter for analyzing any molecular dynamics (MD) trajectory. It serves as a pivotal metric in quantifying the disparities between the protein’s backbone conformation at its initial structural state and its eventual position. By scrutinizing the deviations that emerge during the MD simulation process, we can ascertain the protein’s stability concerning its conformation. Typically, to extract meaningful data, an RMSD value for a macromolecule should fall below the threshold of 3Å. In this current investigation, a comparative study is being carried out between two complexes namely- *SRC*_NAR and *SRC* _Dasatinib. In this scenario, complex *SRC*_NAR exhibited an average RMSD value of 2.4 Å based on the Cα atoms of the protein backbone, as this metric is commonly used to assess overall structural stability during molecular dynamics simulations. Throughout the simulation period the target was stable but during 60–80 ns a peaked fluctuation was observed. Complex *SRC* _Dasatinib exhibited an average RMSD value of 2.3 Å. In this case the target protein *SRC* showed more or less steadiness during the simulation period of 100 ns at 37 °C. These findings suggested that *SRC* _Dasatinib formed comparatively a better complex with melanin with minimal effect exhibited upon binding. MD simulation analysis of 100 ns trajectories is shown in Fig. 9.

#### RMSF

Analyzing the root mean square fluctuation (RMSF) proves to be the most effective approach for examining residue-wise fluctuations within the protein across an MD trajectory. The RMSF plots further indicate the flexibility of Cα-atoms within the protein when it is bound to a Ligand, providing insights into the fluctuation patterns of individual residues or domains within the protein structure. In this current investigation, almost the two complexes showed a similar pattern of fluctuations over the entire 100 ns simulation period as evidenced from the graphical representation. The two complexes namely- *SRC*_NAR and *SRC* _Dasatinib showed an average RMSF value of about 1.02 Å and 1.20 Å respectively. These observations suggest that the target molecule undergoes a similar impact upon forming complexes with the Ligand molecules. The highest peaks of fluctuations are observed primarily within residue ranges 1–10.

### Radius of gyration

The protein’s radius of gyration (Rg) serves as an indicator of its structural compactness. To assess the evolving compactness of the system over time, Rg was computed, where elevated Rg values signify decreased compactness (greater unfolding) accompanied by increased conformational entropy. Conversely, lower Rg values signify heightened compactness, reflecting greater structural stability (more folding). In this current investigation, the two complexes *SRC*_NAR and *SRC* _Dasatinib showed an average Rg value of about 14.58 Å and 13.8 Å respectively. The graphical representations showed pronounced fluctuations in the target molecule *SRC* upon complex formation with NAR and Dasatinib. So, these findings imply a reduction in the target molecule’s compactness, along with heightened conformational entropy and decreased stability. H-bond analysis: Hydrogen bonding serves as a pivotal factor in assessing the binding strength between Ligands and proteins. The count of hydrogen bonds established between the protein and Ligand serves as a reliable indicator of the substantial interactions and overall stability of the complex. The hydrogen bond plot for NAR and Dasatinib revealed an average of approximately 5 and 3 hydrogen bonds, respectively, maintained consistently throughout the 100 ns simulation period. These results imply that NAR shared a strong interaction with the target protein *SRC* than Dasatinib at 37 °C.

#### MM/GBSA analysis

In this current investigation, the approximate binding free energies of the respective complexes are summarized in Table 3. The available evidence suggested that Dasatinib has the potential capability to form a stable protein-ligand complex as it exhibited comparatively higher binding free energy ΔG_bind_ = −62.50 kcal/mol. Out of all interaction types, G_bind_vdW and G_bind_Coulomb emerged as the primary contributors to the average binding energy, whereas GbindSolvGB and G_bind_Covalent energies exerted the least influence on the overall average binding energies.

The results indicate that both NAR and Dasatinib exhibit strong binding affinities towards the *SRC* protein, with Dasatinib showing the highest binding free energy. The primary contributors to the binding energy in these complexes are the van der Waals (vdW) interactions and Coulomb interactions, which are essential for the stability of the protein-ligand complexes. The covalent and solvation energies contribute minimally to the overall binding energy.

**Table 3:** The binding free energy along with other associated energy in the form of MM-GBSA were determined for each complex at 37 °C for 100 ns.

Energies (kcal/mol)	SRC-Naringenin	SRC- Dasatinib
ΔGbind	−58.25	−62.50
ΔGbindCoulomb	−10.35	−12.75
ΔGbindCovalent	3.20	3.50
ΔGbindHbond	−0.78	−0.85
ΔGbindSolvGB	20.15	18.70
ΔGbindvdW	−45.47	−50.30

### Principal component analysis (PCA)

**Fig. 10:**
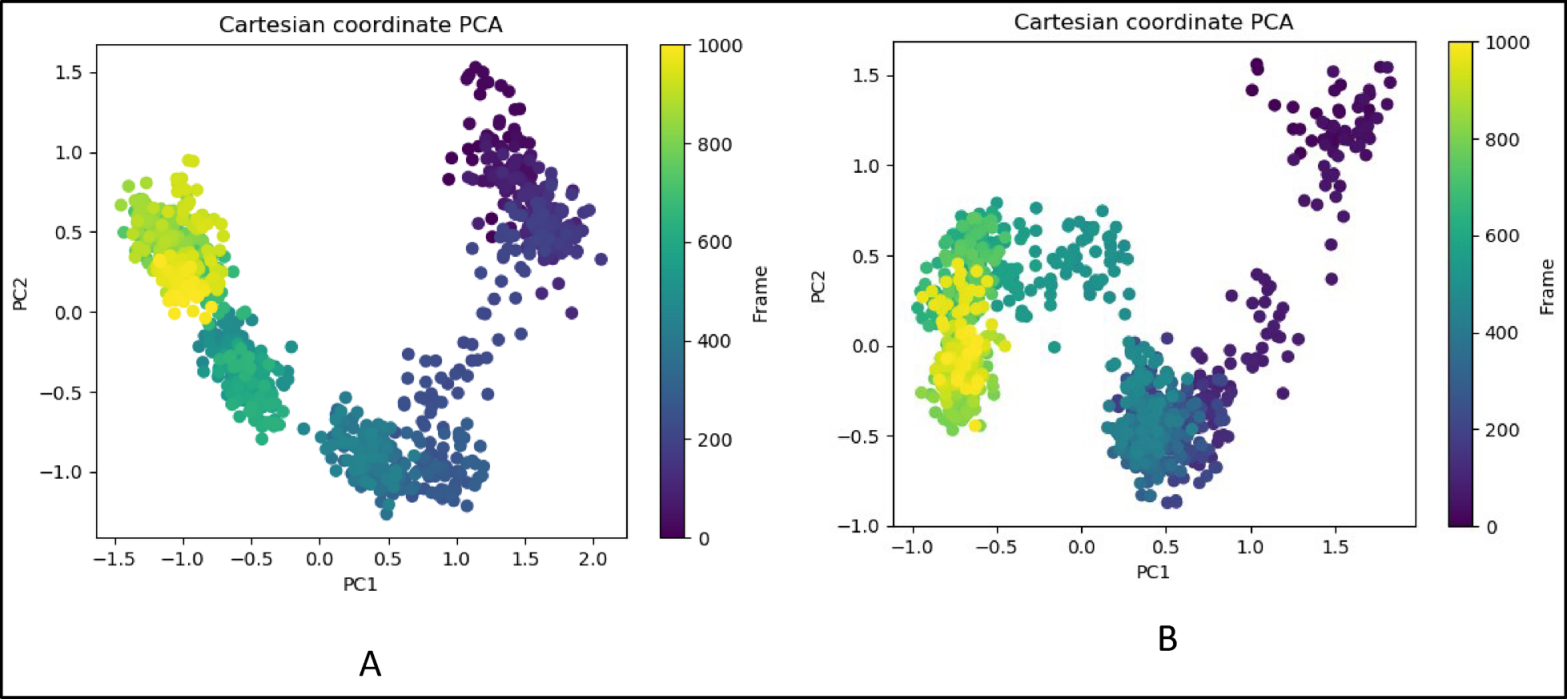
(**A**): PCA plot of *SRC* bound to NAR, (**B**): PCA plot of *SRC* bound to Dasatinib.

The Molecular Dynamics (MD) frames are distributed in various clusters, with the yellow dots representing the last 150 frames for *SRC* bound to (A) NAR, (B) Dasatinib, and (C) Protein_*SRC*. PCA is a statistical technique used to emphasize variation and bring out strong patterns in a dataset. It’s commonly used in molecular dynamics to analyze the motion of molecules over time, reducing the dimensionality of the data while preserving as much of the variation as possible. In this case, the PCA plots are used to visualize the dynamics and conformational changes of *SRC* when bound to different ligands (NAR, Dasatinib, and the protein *SRC* itself). The presence of clusters in these plots suggests that the MD simulations of *SRC*, when bound to the different compounds, exhibit distinct conformational states or dynamics. Each cluster may represent a stable state or a group of similar conformations that the protein-ligand complex adopts over the simulation time. The yellow dots, representing the last 150 frames, likely highlight the ending frames of each simulation, focusing on the final conformational states of the *SRC*-ligand complexes. The distribution of these yellow dots across the clusters can provide insights into the stability and predominant conformations of the complexes toward the end of the simulation. The position of the last 150 frames (yellow dots) in relation to the clusters informs about the convergence of the simulation. If these dots are concentrated in specific clusters, it suggests that the simulation has reached a stable state. If they’re spread out, the system might still be exploring different conformations. In Plot (Fig. 10A), *SRC* bound to NAR exhibits the last 150 frames aggregated into a cluster having the same conformation and converged where PC1 and PC2 exhibited negative correlation. *SRC* bound to Dasatinib exhibits a cluster of the last 150 frames exhibiting a similar pattern of motion and clustered most of the frames out of 150 frames having negative correlation between PC1 and PC2 for ordered orientation and convergence (Fig. 10b). However, the apo-*SRC* exhibits a negative correlation cluster at PC1 and PC2 Eigen vectors (Fig. 10).

### Free energy landscape analysis of SRC with NAR and dasatinib

**Fig. 11:**
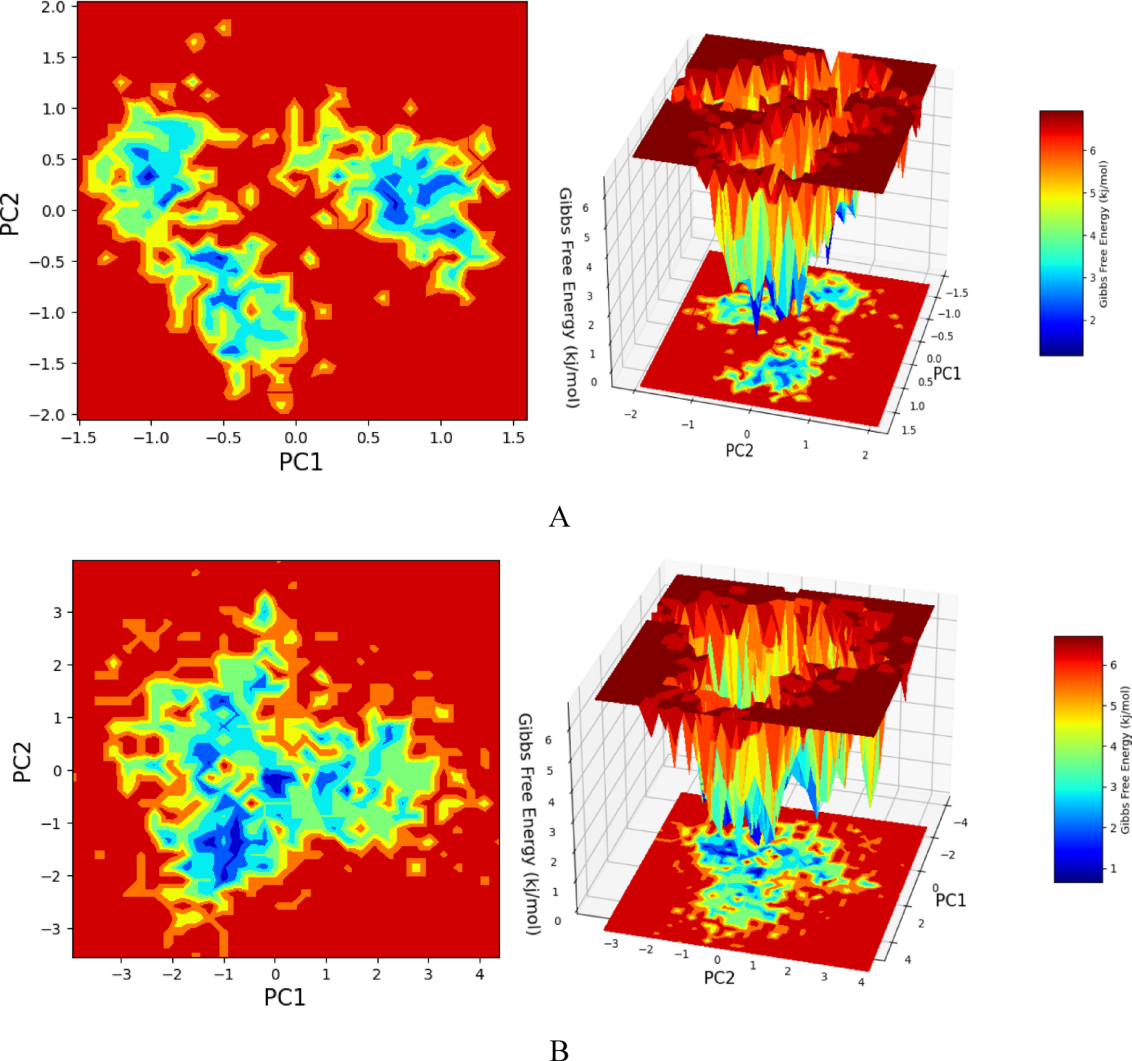
Free energy Landscape (FEL) represented with respect to PC’s for conformational analysis for PC1 vs. PC2, where left panel exhibiting 2D FEL and the clusters of frames. The structures at mid-point have been exhibited with time scale and the right panel exhibiting the well of global minima in 3D representation. (**A**) representing *SRC*_NAR (**B**) *SRC*_Dastanib.

In the free energy landscape analysis, two distinct components—distance among Cα-atoms (PC1) and dihedral angles Φ and Ψ (PC2)—along 2D planes were examined to identify principal motions within the trajectory and critical reaction coordinates. *SRC* bound to NAR displayed a significant cluster of frames in the global minima with minimal local minima, indicating stable protein folding (Fig. 11A). Conversely, the Dasatinib bound state showed an increased number of local minima, signifying higher transition barriers due to aberrant protein folding (Fig. 11B). This behavior suggests that NAR maintains a stable conformation of *SRC*, while Dasatinib induces more dynamic conformational changes. The clustering of trajectories into a single global minimum for NAR -bound *SRC* points to a stable conformational state, whereas the multiple local minima observed for Dasatinib -bound *SRC* reflect the protein’s flexible nature and higher order of transition barriers, which may lead to improper folding. This analysis underscores the differential impacts of NAR and Dasatinib on the structural stability and folding dynamics of *SRC*.

**Fig. 12:**
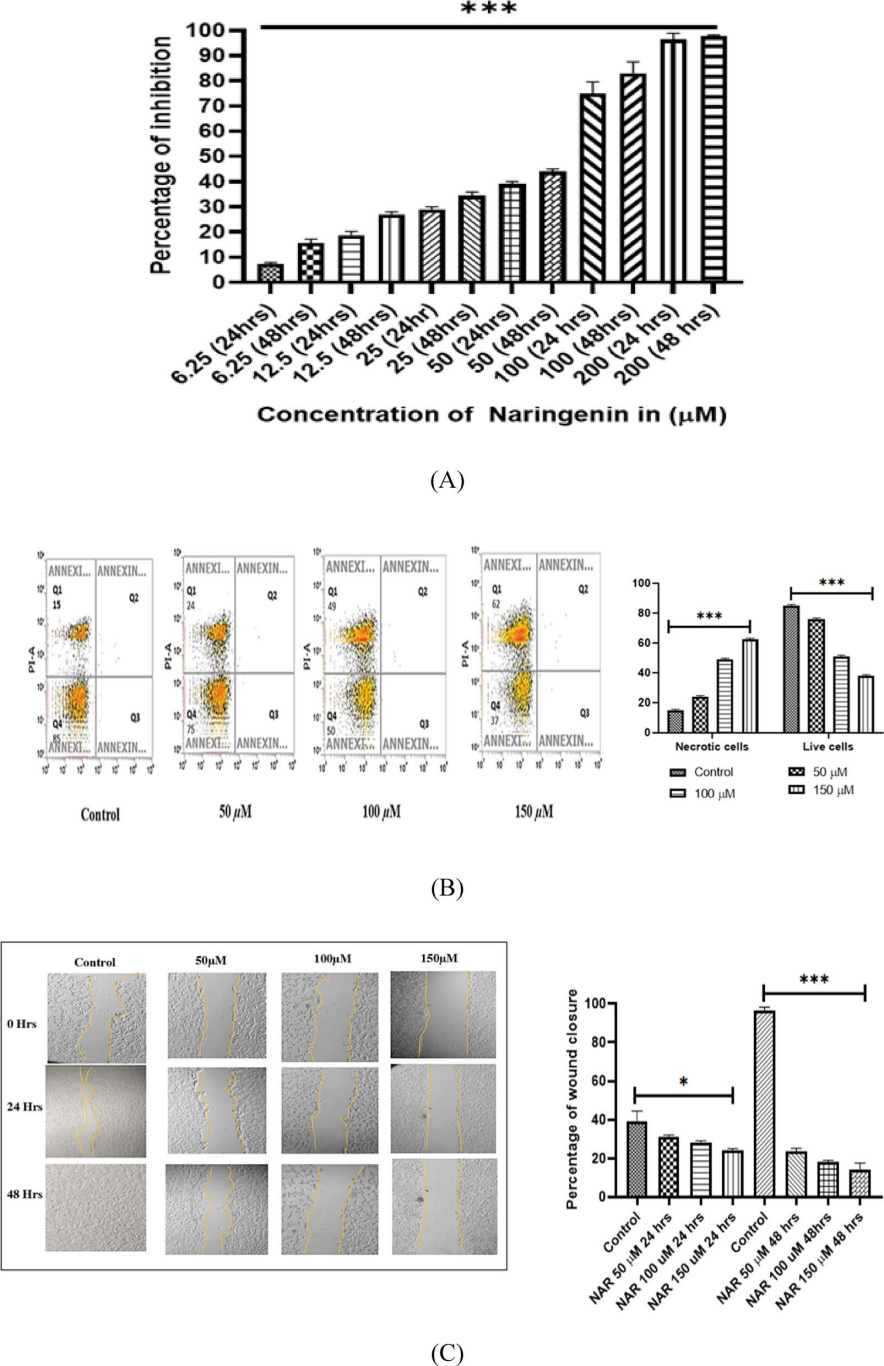

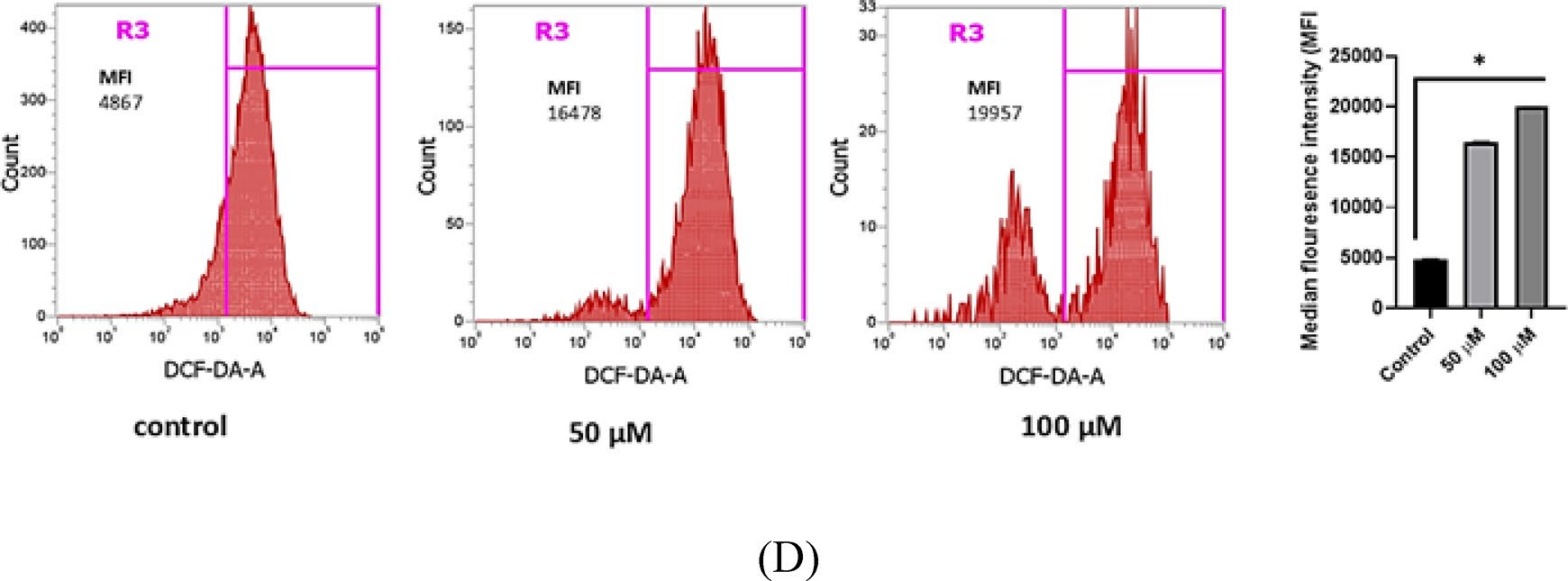
(**A**) Anti-proliferative potential of NAR on human breast cancer cells MCF-7. The percentage of inhibition was found to be both time- and dose-dependent. NAR exhibited an IC_50_ of 88 µM after 24 h of treatment and 45 µM after 48 h, indicating enhanced cytotoxicity with prolonged exposure. Statistical significance is denoted by *** (*p* < 0.0001). Data are presented as mean ± SD from three independent experiments. (**B**) PI flow cytometry analysis of NAR-induced cell death in MCF-7 breast cancer cells. Quadrants: Q1: Necrotic cells (PI⁺); Q2 Late apoptotic cells (PI⁺); Q3: Early apoptotic cells (PI⁻); Q4: Live cells (PI⁻). Data represent mean ± SD from three independent experiments. *p < 0.0001. (**C**) In left, effect of NAR on MCF-7 cell migration assessed by wound healing assay. Representative phase-contrast images showing the effect of NAR at different concentrations (50 µM, 100 µM, and 150 µM) on wound closure in MCF-7 breast cancer cells over 48 h. In right graph, percentage of wound closure in MCF-7 cells, Values are presented as Mean ± SD, and the data is indicative of three separate studies. *P < 0.05, ***P < 0.0001. The wound area in MCF-7 cells at each time point is delineated by the yellow Lines. While the initial wound borders are visible at 0 h, the wound closure is shown at 24 and 48 h when the cells migrate into the wound area. (**D**) ROS production in MCF-7 cells induced by NAR, evaluated through DCFDA staining and flow cytometry. The flow cytometry histograms illustrate ROS generation in MCF-7 cells following treatment with 50 µM and 100 µM NAR for 24 h. Prior to analysis, cells were incubated with 0.5 µM DCFDA for 25 min. ROS levels were determined based on median fluorescence intensity (MFI) in region R3 (highlighted in pink). From left to right: Control (MFI = 4867), 50 µM NAR (MFI = 16478), and 100 µM NAR (MFI = 19957). Data are expressed as mean ± SD from three independent experiments. *p < 0.05.

### In-vitro analysis

We validated its role in invitro conditions using MCF-7 breast cancer cells by performing 3-(4,5-dimethylthiazol-2yl)−2,5-diphenyl-2 H-tetrazolium bromide (MTT) and found that NAR inhibited the cellular proliferation in a dose and time dependent manner as shown in Fig. 12A, suggesting the antiproliferative potential of NAR against breast cancer cells^78^. To further validate its cell death potential, we performed a flowcytometric basis test, PI staining and found that cell death was concentration dependent. Live cell populations were decreased from 85% to 37% and necrotic cell population was increased from 15% to 62% as shown in Fig. 12B.

Next, we investigated the effect of NAR on cellular migration, and for this wound healing assay was performed. The objective was to evaluate the impact of NAR on the migration MCF-7 breast cancer cells, which is considered important metastatic potential of cancer cells. This assay aims to determine whether NAR enhances or restricts the cellular migration and results revealed the concentration dependent response on wound closure suggesting its role as anti-metastatic candidate against breast cancer MCF-7 cells. The Fig. 12C displays the wound closure at 0, 24 and 48 h of the treated cells against the control. To investigate its potential to triggers apoptosis ROS generation, a DCFDH-DA assay was conducted. The results demonstrated a significant elevation in ROS levels, as indicated by an increase in median fluorescence intensity (MFI) in a concentration-dependent manner. The highest MFI value of 15,551 was observed at 100 µM, as depicted in Fig. 12D.

## Discussion

Recent years have seen an upward trend in the integration of computational systems biology with experimental approaches. This has opened up new opportunities for drug discovery^10,11^. However, despite these advances, our understanding of the underlying mechanisms of natural medicines remains limited, and comprehending the intricate interactions among their complex components is still challenging. Network pharmacology is an emerging discipline that combines computational systems biology and pharmacology^33^. In recent years, it has been widely applied in drug discovery and pharmacological mechanisms research^34^.

In this study, we aimed to investigate the potential mechanisms of NAR in the treatment of breast cancer. By using databases like OMIM, CTD and GeneCards databases using “Breast Cancer” as the keyword and based on the GIFT (GeneCards Inferred Functionality) score of > 50, we systematically screened the components and targets of NAR, 29 active components and corresponding targets were selected. A total of 1,075 Gene targets related to breast cancer and 114 targets associated with naringenin (NAR) were collected from various databases. Among them, 62 targets were found to be common to both categories, with 43 of these predicted to possess a druggability score exceeding 0.5. Using Cytoscape 3.8.0, we constructed a drug-target network and NAR with high degree value in the network. As predicted, NAR has been shown to have anti-breast cancer effect^64,79,80^. Regarding, naringenin (NAR), chemically known as 5,7-dihydroxy-2-(4-hydroxyphenyl) 2,3-dihydrochromen-4-one is hydrophobic citrus flavonone (type of flavonoid), which belongs to the family vitamin P^81^.

Considering that 62 intersection targets are potential anti-breast cancer targets of NAR, we constructed a PPI network using 62 intersection targets. The results of PPI showed that *SRC*, *PIK3CA*,* BCL2*,* and ESR1* exhibited higher degree values. In order to accurately screen core targets, we performed GO and KEGG analysis on the intersecting targets, and the results of GO analysis indicated that the target genes were involved in biological functions such as protein phosphorylation, regulation of programmed cell death and regulation of intracellular signalling transduction. Moreover, the results of KEGG enrichment analysis indicated that NAR may exert its therapeutic effects on breast cancer through multiple signal pathways, such as prolactin signalling pathway, HIF-1 signalling pathway, breast cancer, MicroRNAs in cancer, Proteoglycans in cancer, Hepatocellular carcinoma, Rap1 signaling pathway, PI3K-Akt signalling pathway, Chemical carcinogenesis reactive oxygen species and pathways in cancer. We found that *SRC*, *PIK3CA*,* BCL2*,* and ESR1* showed higher degree values in the PPI network, and enriched in the prostate cancer signal pathway. Therefore, *SRC*, *PIK3CA*,* BCL2*,* and ESR1* may be the core targets for the treatment of breast cancer. Previously studies have shown that these three target proteins (*SRC*, *PIK3CA*,* BCL2*,* and ESR1*) play an important role in the occurrence and development of breast cancer.

*SRC* is a proto-oncogene which plays a pivotal role in the regulation of cellular proliferation and embryonic development. This gene encodes a tyrosine-protein kinase protein, c-*SRC* kinase can phosphorylate to decrease its activity and any mutation in this gene could promote the progression of cancer. Additionally, *SRC* is the part various signalling pathways which regulates gene transcription, cell cycle, immune response, apoptosis, migration, and transformation of cells^82^. *PIK3CA* Gene encodes phosphatidylinositol 3-kinase which is composed of an 85 kDa regulatory subunit and a 110 kDa catalytic subunit. The protein encoded by this gene represents the catalytic subunit, which uses ATP to phosphorylate *PtdIns*,* PtdIns4P* and *PtdIns* (4,5) P2. This gene has been found to be oncogenic and has been implicated in cervical cancers^83^.

*BCL2* gene encodes an integral outer mitochondrial membrane protein that inhibits the programmed cell death of cells like lymphocytes. Constitutive overexpression of *BCL2* has been linked to the progression certain cancers like follicular lymphoma especially when *BCL2* translocate to IG heavy chain locus. It also regulates cell death by controlling the mitochondrial membrane permeability^84^.

The identification of the expression profile of *SRC* Gene in BC was conducted through the utilisation of bioinformatics methodologies. To accomplish this, various computational tools such as UALCAN, TIMER 2.0, Gepia2, and GenExMiner were utilized. For instance, *SRC* expression was analysed using UALCAN and it has been revealed that in the breast cancer (BC) is significantly overexpressed. However, the expression profile of *SRC* in several malignancies was analysed using TIMER 2.0 and GEPIA2, and the results showed that *SRC* is strongly elevated in multiple cancers, including Breast Cancer (Figs. 2 and 3). While network pharmacology offers valuable insights into complex drug-disease interactions, it has inherent limitations. Network pharmacology faces limitations such as bias in target prediction databases, which often prioritize well-characterized proteins, leading to the underrepresentation of novel or less-studied targets. This skews results toward known pathways and may hinder the identification of truly novel interactions. Additionally, false positives are common due to incomplete or noisy interaction data, resulting in predicted associations that may lack biological validity. Protein-protein interaction models can also overpredict links, especially when pathway definitions are unclear, reducing specificity and interpretability^85,86^.

Molecular docking is of immense value in studying molecular interactions and allows the prediction of binding patterns and affinities of molecules. To explore the amino acid binding sites of NAR in the treatment of breast cancer, NAR was screened through network pharmacology, as well as the core target proteins (*SRC*, *PIK3CA*,* BCL2*,* and ESR1*) through PPI and bioinformatics technology. We also studied precise amino acid sites and 3D/2D spatial structure through computer modelling. The molecular docking results were consistent with the predictions of network pharmacology. NAR had good binding affinity with the core targets as was evident with its less than − 5 kcal/mol binding energy of molecular docking. (when the binding energy of molecular docking is less than − 5 kcal/mol, it is considered that there is a good binding affinity between the ligand and the protein)^87^. The binding affinity between NAR and *SRC* gene (−9.21 kcal/mol) was the highest, representing the most stable binding.

NAR has been found to induce apoptosis and causes cell cycle arrest in a number of cancer cell lines including MDA-MB-231 cell line^64^. It also inhibits cellular migration, causes cell cycle arrest and induces apoptosis in human lung cancer, A549 cell line^88^. It has also been found to induce autophagy, and apoptotic cell death via the ROS-Production and endoplasmic stress in in osteosarcoma cells^89^. NAR inhibited cellular proliferation, and migration indicating its anti-metastatic potential. Reactive oxygen species (ROS) play a crucial role in regulating cellular functions such as proliferation, apoptosis, and autophagy. Elevated ROS levels can induce oxidative stress, potentially cause DNA damage and triggering apoptotic signaling pathways. The findings demonstrate that treatment with NAR significantly enhanced ROS production, as reflected by an increase in median fluorescence intensity (MFI). Further investigation using ROS scavengers like N-acetylcysteine (NAC) could help clarify its involvement in cell death mechanisms. However Further insights to its molecular mechanism could provide the in-depth analysis of the compound whether to use it as a mano therapeutic agent or in adjuvant to the already chemotherapeutic agents. The bioavailability and pharmacokinetic profile of Naringenin (NAR) are critical determinants of its therapeutic potential. Evaluating its efficacy in combination with conventional chemotherapeutic agents may offer valuable insights into potential synergistic interactions and enhanced anticancer effects.

## Conclusion

This study provides a comprehensive molecular insight into the anti-breast cancer potential of Naringenin (NAR) through an integrative approach combining network pharmacology, molecular modeling, and in-vitro analyses. The findings reveal that NAR is predicted to exert potential inhibitory effects on breast cancer by targeting key oncogenic proteins, particularly *SRC*, *PIK3CA*,* BCL2*,* and ESR1*, as suggested by strong binding affinities in molecular docking studies and stable interactions observed in molecular dynamics simulations. Functional enrichment analysis further indicates that NAR modulates critical signaling pathways, including the PI3K-Akt and MAPK pathways, which are crucial in cancer cell survival and proliferation. In-vitro studies demonstrated that NAR effectively suppresses breast cancer cell proliferation, induces cell death via necrosis, reduces migration, and enhances reactive oxygen species (ROS) generation, further validating its anticancer potential. Overall, the study highlights NAR as a promising natural therapeutic agent with potential implications for developing novel *SRC*-targeted inhibitors in breast cancer treatment.

## Data Availability

The data is available and will be shared upon a reasonable request to Suhail Ahmad Mir (suhailmir675@gmail.com). & Ghulam Nabi Bader (gnbader@kashmiruniversity.ac.in).
